# Nintedanib and a bi-specific anti-VEGF/Ang2 nanobody selectively prevent brain metastases of lung adenocarcinoma cells

**DOI:** 10.1007/s10585-020-10055-x

**Published:** 2020-09-12

**Authors:** Bogdana Kovalchuk, Anna S. Berghoff, Matthia A. Karreman, Katharina Frey, Manuel Piechutta, Manuel Fischer, Julia Grosch, Sabine Heiland, Michael O. Breckwoldt, Frank Hilberg, Wolfgang Wick, Frank Winkler

**Affiliations:** 1 Neurology Clinic and National Center for Tumor Diseases, University Hospital Heidelberg, Im Neuenheimer Feld 400, 69120 Heidelberg, Germany; 2 Clinical Cooperation Unit Neurooncology, German Cancer Consortium (DKTK), German Cancer Research Center (DKFZ), Heidelberg, Germany; 3 Department of Neuroradiology, University Hospital Heidelberg, Heidelberg, Germany; 4 Department of Pharmacology, Boehringer Ingelheim RCV GmbH & Co KG, Vienna, Austria; 5 Division of Oncology, Department of Medicine I, Medical University of Vienna, Vienna, Austria

**Keywords:** Lung adenocarcinoma, Cancer prevention, Anti-angiogenic drugs, VEGF-A, Ang-2, Brain neoplasms, Xenograft metastasis model, Therapeutic IgG

## Abstract

Brain metastases (BM) are an ever-increasing challenge in oncology, threatening quality of life and survival of many cancer patients. The majority of BM originate from lung adenocarcinoma, and stage III patients have a risk of 40–50% to develop BM in the first years of disease onset. As therapeutic options are limited, prevention of their occurrence is an attractive concept. Here we investigated whether Nintedanib (BIBF 1120), a tyrosine kinase inhibitor (TKI) targeting the VEGF pathway approved for lung adenocarcinoma, and the dual anti-VEGF-A/Ang2 nanobody BI836880 have the potential to prevent BM formation. A mouse model of brain metastasis from lung adenocarcinoma was used in which tumor cells were injected intracardially. Metastases formation occurred inside and outside of the brain and was followed by MRI, IVIS, and immunohistochemistry. BM were reduced in volume and number by both Nintedanib and the dual anti-VEGF-A/Ang2 nanobody, which translated into improved survival. Both compounds were able to normalize cerebral blood vessels at the site of brain metastatic lesions. Extracranial metastases, however, were not reduced, and meningeal metastases only partially. Interestingly, unspecific control IgG also lead to brain vessel normalization and reduction of brain and meningeal metastases. This data indicates a brain-specific group effect of antiangiogenic compounds with respect to metastasis prevention, most likely by preventing an early angiogenic switch. Thus, Nintedanib and BI836880 are promising candidates for future BM preventive study concepts in lung adenocarcinoma patients.

## Introduction

Brain metastases (BM) have an increasing incidence [1–3] and are associated with high morbidity and mortality, affecting neurological function and quality of life, with a mean overall survival of affected patients of a few months only. Many patients suffering from solid cancers are at high risk of developing BM during the course of their disease. BM occur most frequently in lung adenocarcinoma, where up to 50% of stage III patients (locally advanced) develop BM within 24 months after “definitive” treatment with surgery, radiation, and chemotherapy [4, 5].

The treatment of established, clinically relevant BM is complicated by the fact that the disease is generally multifocal in nature. Moreover, the brain is a difficult, delicate organ where many locally aggressive and systemic therapies are not possible or not effective [3]. One major issue is that most chemotherapeutics fail to pass the blood brain barrier (BBB) and blood tumor barrier in sufficient concentrations [6]. Targeted therapies, however, can be very effective against established BM from different entities, such as BRAF and MEK inhibitors in melanoma, EGFR and ALK inhibitors in lung adenocarcinoma, and immune checkpoint inhibitors in melanoma and lung cancer [3, 7–10]. There is also some promising clinical data for antiangiogenic drugs, particularly bevacizumab, with indications for activity against BM, additional beneficial anti-edema effects, and reduction of radiation-induced necrosis [11–18].

Considering the difficulties of BM treatment, it appears logical that their prevention must be a prime goal of future therapies. Especially patients with a high risk to develop brain metastasis (HER2+ or triple-negative breast cancer, melanoma, small-cell lung cancer and stage III/IV non-small-cell lung cancer (NSCLC) [3]) would benefit from such a preventive intervention. Prophylactic whole-brain radiation therapy has already proven to be effective in reducing BM [19, 20], although associated with significant neurotoxicity [19–22]. BM prevention by a non-neurotoxic treatment, ideally a systemic drug that is well tolerated even with long-term administration, is therefore an exciting possibility that however is still in need for a full pre-clinical characterization and clinical validation. Conceptually, the most promising biological approach would be to target BM initiating cancer cells at very early time points of the brain metastatic cascade, e.g. by preventing the mandatory and VEGF-dependent angiogenic switch during micrometastases proliferation in lung adenocarcinoma [3, 15, 23]. The anti-VEGF-A antibody bevacizumab was shown to prevent BM in a lung adenocarcinoma mouse BM model [23], and the retrospective analysis of the AVAIL trial revealed a reduction of BM as first site of relapse with bevacizumab treatment [15]. Moreover, in a prospective clinical trial of stage IV lung adenocarcinoma with mutated EGFR, the addition of bevacizumab to the standard tyrosine kinase inhibitor (TKI) treatment increased progression free survival and reduced BM formation [24]. It remains unclear whether the brain-specific preventive effect seen in both these studies was an explicit effect of bevacizumab, or rather due to a class effect of antiangiogenic drugs. For a clinical BM prevention concept, oral drugs that can be taken over years in an outpatient setting might be more feasible. In a phase 3 multicenter double-blind randomized trial (LUME-Lung 2 [25]) the oral anti-VEGF pathway TKI Nintedanib has prolonged survival in relapsed or refractory advanced NSCLC.

In this study, the two antiangiogenic compounds Nintedanib and the new dual anti-VEGF/Ang2 nanobody BI836880 were therefore tested for their ability to inhibit BM development and growth, reduce development of extracranial metastases, and prolong survival. For this purpose, a mouse model was selected that reliably reflects the human disease, including frequent development of BM that are relevant for the clinical course after systemic tumor cell inoculation [15, 23].

## Material and methods

### Murine brain metastasis model

The brain seeking subline PC14 -PE6 pGF1 Br4 of the human lung adenocarcinoma cell line PC14-PE6 was used to model brain metastasis formation in immunodeficient NOD/SCID mice, as described by Ilhan-Mutlu et al. [15]. To receive an even higher brain affinity and number of BM, two additional reinjections of successfully brain-metastasized cells, as described before [15], were performed and the PC14-PE6 pGF1 Br4 cell line was obtained from the previously used PC14-PE6 pGF1 Br2 cell line. This so called “brain training” does increase the chance of metastatic cells to form metastases in the brain and does not primarily inhibit metastasis formation to other organs [26].

Briefly, PC14-PE6 pGF1 Br4 cells were trypsinized (Gibco, Life Science Technologies, cat. no.: 25200-056), washed, counted and resuspended in PBS (cat. no: D8537, Sigma Life Sciences) in a final concentration of 5 × 10^6^ cells/mL. 8-week-old male NOD/SCID mice were anesthetized with a xylazine-ketamine-injection (intraperitoneal injection of 100 µL NaCl-solution containing 1 mg/mL of xylazine (Rompun 2%, 20 mg/mL, Bayer) and 15 mg/mL ketamine (100 mg/mL, WDT)). Thereafter 5 × 10^6^ PC14-PE6 pGF1 Br4 cells (100 µL) were singularized through a filter tube (BD-Falcon, BD Biosciences, cat. no: 352235) and injected in into the left cardiac ventricle with a 30G needle. Through blood-circulation tumor cells are then distributed into the organs. All animals were handled according to the German animal protection law (Approving institution: Regierungspräsidium Karlsruhe).

Cells were cultured in DMEM (PAN Biotech, cat. no: P04-03600, 500 mL) containing 4.5 g/L glucose, sodium pyruvate, 3.7 g/L NaHCO3 without l-glutamine supplemented with 10% heat-inactivated FBS (Sigma-Aldrich, cat. no: F7524), 1 mL penicillin/streptomycin (Sigma-Aldrich, cat. no: P4333), and 5 mL of Glutamax (Gibco, Life Science Technologies, cat. no.: 25200-056) in a humidified atmosphere of 10% CO2 at 37 °C. They were passaged via trypsinization (Gibco, Life Sciences, cat. no: 25200-056) when reaching 90% of confluence. Transduced with a pGF1-CMV reporter, the used cells express both, copGFP and *firefly* luciferase. Thus, FACS sorting of GFP-expressing cells was performed (on FACSAria1, BD Biosciences) prior to cell expansion for injection. Furthermore, cell line authenticity was confirmed using a Multiplex human cell line authentication test, which is provided by Multiplexion.

### Treatment protocol

To evaluate different antiangiogenic compounds, mice were randomized to four separate intervention groups with 12 mice per group (control IgG group n = 14). Treatment started one day prior to heart injection to ensure full BM preventive activity and was always adapted to body weight.

The first group received daily treatment with Nintedanib (BIBF 1120, Boehringer-Ingelheim) in comparison to its control group, receiving 200µL of carrier solution (0,5%-Hydroxyethylcellulose, cat. no.: 822068, Merck) only. Nintedanib is a triple angiokinase inhibitor blocking VEGFR, PDGFR and FGFR kinase activity and was shown to reduce vessel density and vessel integrity in human tumor xenografts [27]. It was solved in 0,5%-Hydroxyethylcellulose (final concentration 5 mg/mL) and applied via oral gavage in a dosage of 50 mg/kg (ca. 200 µL per mouse).

The third group was treated every 3rd day with a combined anti-VEGF and anti-Ang2 nanobody (BI836880, MW appr. 40.7 kDa; obtained by Boehringer-Ingelheim) in contrast to its respective control group (fourth group), which received a control antibody (InVivoMAb rat IgG2a isotype control, MW 150 kDa; BioXCell) of equal dosage, frequency and concentration. Nanobody and control antibody were solved in sterile PBS (cat. no: D8537, Sigma Life Sciences) reaching a concentration of 2.615 mg/mL, their application dose was 15 mg/kg (100–150µL per mouse).

### In vivo bioluminescence imaging (IVIS)

Metastasis development was monitored by in vivo bioluminescence imaging (IVIS Lumina Series III Imaging system, PerkinElmer) on day 1 (baseline imaging), day 14 and day 28 after tumor cell injection. For image acquisition the mice received an intraperitoneal injection of Luciferin (Luciferin substrate cat. no.: 5306500001, Calbiochem; dosage: 150 mg/kg; stock solution: 30 mg/mL in H_2_O; application volume: 100–150 µL). After 3 min of incubation the animals were sedated with 5% isoflurane and then transferred to the imaging chamber with 2% isoflurane and 37 °C. Imaging was started 10 min after Luciferin injection using the XFOV-24 lense and an exposure time of 180 sec (medium bining, 1.2 F/Stop, minimum target count luminescent: 10,000). Images were taken from the ventral as well as from the dorsal view.

### In vivo cranial MRI

For more precise in vivo evaluation of intracranial metastases formation, cranial MRI (cMRI, 9.4 T, Bruker Topspin 9/20) after Gadolinium contrast administration was performed on day 26 after intracardial tumor cell injection. Mice were sedated with 3% isoflurane and kept under anesthesia at 0.5–1.5%. Constant body temperature was maintained at 37 °C by a heating plate. During imaging respiration was surveilled using an external breathing surface pad (in house development, LabVIEW program, National Instruments Corporation). A dose of 0.2 mmol/kg i.v. gadodiamide (Omniscan; Nycomed) was given to each animal and standard T1-w and T2-w images were acquired. For quantification of tumor volumes, tumors were manually segmented on T1-w images using the Fiji software (general public license) [28].

### Follow up and organ preservation

To prevent confounding and observer bias, mice of different intervention groups were hold together in common cages and were distinguished by small ear punches, visible only at a very close look. Daily control for adverse events, including neurological symptoms, poor outer appearance and weight loss of more than 20%, was performed. At the occurrence of one of the latter the animals were sacrificed to prevent suffering and their lifetime was documented for survival analysis. A left cardiac perfusion with PBS (cat. no: D8537, Sigma Life Sciences) and, subsequently, 4.5% paraformaldehyde (Roti-Histofix, cat. no: 22135, ROTH) was performed. After 1 h of paraformaldehyde fixation at room temperature, the brain tissue was incubated in 30% sucrose solution (cat. no: 84097-1 KG, Sigma Life Sciences, diluted in PBS) at 4 °C for 24 h and preserved at − 80 °C, embedded into optimal cutting temperature medium (Roti-Histofix, cat. no: 22135, ROTH).

### Histology and immunostaining

To prepare slides for histology, brain tissue of 6 mice per group (randomly chosen to prevent observer bias) was cut into 12 µm thick coronary cryo-sections with a layer distance of 1000 µm (cryostat Leica CM3050 S). Vascular basement membrane staining was performed as described previously [15, 29] using an anti-collagen-IV primary antibody (1:200, rabbit anti-collagen type IV, cat. no.: AB756P, Merck) and a fluorescent secondary antibody (1:400, goat anti-rabbit AlexaFlour 546, cat. no.: A11010, life). Briefly, slides were dried under air flow (10 min), washed with ice-cold acetone (1 × 10 min) following PBS (3 × 5 min) and then delineated with an invisible fat marker (Dako Pen, cat. no: 52002). After incubation with blocking buffer (1 × 30 min; blocking buffer = 10% donkey serum in TBST buffer (Tris-buffered saline, 0.1% Tween 20 cat.no.: 52194-1 g, Sigma) the primary antibody was applied, and the samples were incubated at 4 °C overnight. The next day they were washed with PBS (3 × 5 min) and covered with secondary antibody for 1 h. Finally, the slides were washed with PBS (3 × 5 min) and shielded with a drop of Vectashield mounting medium with DAPI (Vector Laboratories, cat. no: H-1500) and a cover slip before their storage at 4 °C.

Images for the analysis of metastatic foci were acquired by a brightfield slide scanner (Zeiss Axio Scan. Z1, magnification: × 20, tile-scans of the whole section area), whereas images for vascular basement membrane assessment were taken confocally (Zeiss LSM 710 ConfoCor 3, magnification × 40). Thereby, high resolution images of three different maximally vascularized areas were recorded, both inside and outside of metastatic foci. All images were taken with identical acquisition settings and GFP, DAPI and Alexa Flour 546 signal was detected.

### Image analysis

In histology analysis, metastatic events were counted manually, using the ZEN lite software (Zeiss, blue edition). GFP positive metastatic foci were easily detectable by fluorescence microscopy [23, 30]: To quantify the thickness of collagen-IV positive vascular basement membrane 4 × 4 grids were superimposed onto 300 µm x 300 µm large confocal images as described previously [29]. Diametral thickness was measured wherever Alexa Flour 546 positive structures and grid lines intersected. If less than 6 intersections occurred, 6 × 6 grids were used. Area measurements were performed with the Fiji software measurement tool, after converting the images to RGB and selecting the area of GFP positive foci with the color threshold tool. Non-parenchymal meningeal metastases were excluded from area measurements by the selection tool. As scaling information was changed when transferring the images from ZEN to Fiji, pixel size properties in Fiji were adapted to fit the actual scale parameters recorded in ZEN.

Original cMRI data were converted into Fiji stacks and metastasis volume was measured using the segmentation editor and 3D manager plugins. Metastases were depicted manually on T1-w images by their hyperintense spherical character. For a separate investigation of meningeal and parenchymal metastases, lesions with any connection to the meningeal space were defined as meningeal metastases in contrast to parenchymal metastases, which needed to be surrounded by brain parenchyma only.

For IVIS evaluation, Living Image Software Version 4.4 (PerkinElmer) was used and time-sequences of each mouse were created. Consequently, total photon flux (p/s) was quantified, using the baseline image of day 1 to define background ROIs. For each mouse, a region of interest (ROI) was defined, covering the extracranial part of the body from the ears to the tail root. Ventral and dorsal view were quantified separately.

### Statistical analysis

Statistical analysis was performed using GraphPad Prism Software. Survival time was analyzed with Kaplan–Meier survival curves and Gehan-Breslow-Wilcoxon tests. Development of brain metastases was compared by Fisher’s exact test. Differences in number and volume of metastases were tested with the Mann–Whitney-U test, and this test was also used to determine extracranial photon flux in IVIS and the thickness differences of the vascular basement membrane in immunohistochemistry.

## Results

### Nintedanib and an anti-VEGFA/Ang2 nanobody prevent brain metastases and improve survival

The potential of both Nintedanib, a clinically approved TKI for lung adenocarcinoma, and an investigational dual anti-VEGF/Ang2 nanobody (BI 836880) to prevent the occurrence of clinically relevant BM was investigated. A brain-seeking PC14-PE6 human lung adenocarcinoma subline (PC14-PE6 pGF1 Br4) was selected, which robustly generates a sufficient number of BM for analysis, but to a lesser extent also extracranial metastasis in lung, bone and other organs. Importantly, the results obtained with this cell line demonstrated a high level of concordance between mouse studies and analysis of patient data with respect to patterns of brain and systemic metastasis development [15, 23]. Mice were divided into four treatment groups, receiving either Nintedanib vs. control gavage, or anti-VEGF/Ang2 nanobody vs. control antibody treatment during the metastatic process.

**Fig. 1:**
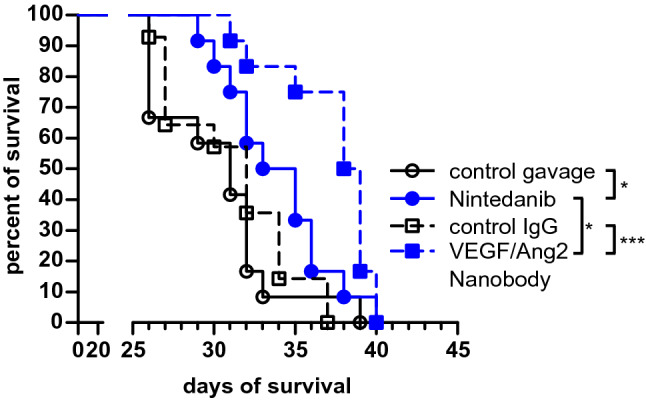
Nintedanib and VEGF/Ang2 nanobody prolong animal survival. Kaplan–Meier survival curves of the four treatment groups receiving control gavage, Nintedanib, control IgG, or VEGF/Ang2 nanobody. Median survival: 32 days; n = 12 mice per group, except of control IgG group n = 14. *p < 0.05 and ***p < 0.001 Gehan–Breslow–Wilcoxon test

**Fig. 2:**
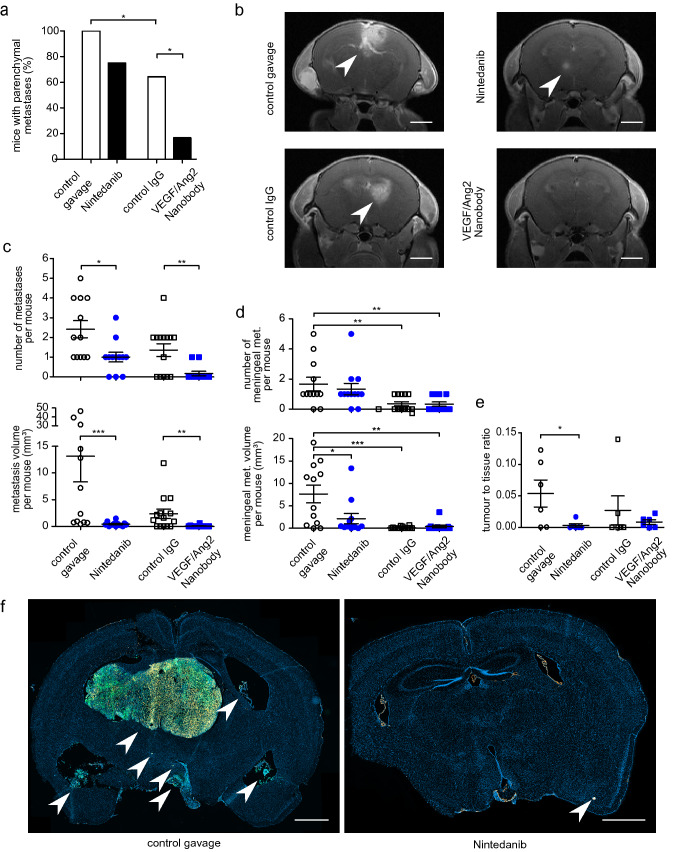
Nintedanib and anti-VEGF/Ang2 nanobody prevent brain metastases formation. 9.4 T MRI after Gadolinium contrast administration was performed on day 26 after intracardial tumor cell injection. **a** Bar chart illustrating the reduced percentage of mice with metastases, detectable in cMRI. **b** Representative T1-w cMRI images depicting larger intracranial metastases in the control groups, indicated by arrowheads. scale bar = 2 mm. **c** Scatter plots showing the reduction of number and volume of cranial metastases in cMRI. **d** Scatter plots depicting the number and volume of meningeal metastases in cMRI **e** Quantification demonstrating a higher histological tumor–tissue ratio in control mice at their time of death. **f** Representative histological slices with higher number and size of PC14-PE6 pGF1 Br4 metastases in the control group. Fluorescent staining: DAPI (blue) = nucleus, GFP (green) = PC14-PE6 tumor cells, Alexa Flour 546 (orange) = collagen-IV positive vascular basement membrane. Arrows indicate GFP-positive metastatic lesions. Scale bar = 1000 µm. Mean values with standard errors of the mean are shown. *p < 0.05, **p < 0.01, ***p < 0.001, Mann–Whitney-U test. (Color figure online)

Both Nintedanib and the anti-VEGF/Ang2 nanobody prolonged the survival of metastases-bearing animals (Fig. 1; p = 0.0246 for Nintedanib, p = 0.0003 for anti-VEGF/Ang2 nanobody, Gehan–Breslow–Wilcoxon test), with the anti-VEGF/Ang2 nanobody being even more effective than Nintedanib (p = 0.017, Gehan–Breslow–Wilcoxon test). First neurological symptoms occurred on day 26 after tumor cell injection, mostly followed by a rapid disease progression. The maximum survival was 40 days, when the last animals were sacrificed according to animal regulations. Evaluation with high field 9.4 T cranial MRI (cMRI) on day 26 revealed that 75.0% (9/12) of mice with Nintedanib treatment developed detectable brain parenchymal metastases (BM), in contrast to 100% (12/12) in their matched control (Fig. 2a). Strikingly, only 16.7% (2/12) of VEGF/Ang2 nanobody treated mice developed radiologically detectable BM, in comparison to 64.3% (9/14) in the control antibody group (p = 0.0214, Fisher’s exact test), supporting the BM-preventive potential of nanobody-treatment. Interestingly, when directly comparing the control groups, the control IgG group had significantly less BM than the group receiving control carrier solution via oral gavage (p = 0.0425).

### Nintedanib and nanobody reduce metastasis formation specifically in the brain

We further aimed to investigate if prolonged overall survival was not only a result of the reduced number of mice developing clinically relevant brain metastases, but also a decrease in number and size of the BM. Using Gadolinium contrast cMRI at day 26 post intracardial tumor cell injection, metastatic lesions were detected and quantified (Fig. 2b). Both Nintedanib and the anti-VEGF/Ang2 nanobody significantly reduced the number (p = 0.0123 for Nintedanib and p = 0.0059 for nanobody, Mann–Whitney-U test) and volume (p = 0.0006 for Nintedanib and p = 0.0055 for nanobody, Mann–Whitney-U test) of brain parenchymal metastases (Fig. 2c). Furthermore, Nintedanib also decreased the volume but not the number of meningeal metastases (Fig. 2d; p = 0.0459, Mann–Whitney-U test), while the anti VEGF/Ang2 nanobody did not (p = 0.9758, Mann–Whitney-U test). Again, number and volume of meningeal metastases were significantly reduced, when comparing the unspecific IgG control with the oral gavage control group (Fig. 2d, p = 0.0057 (number) and p = 0.0007 (volume), Mann–Whitney-U test).

**Fig. 3:**
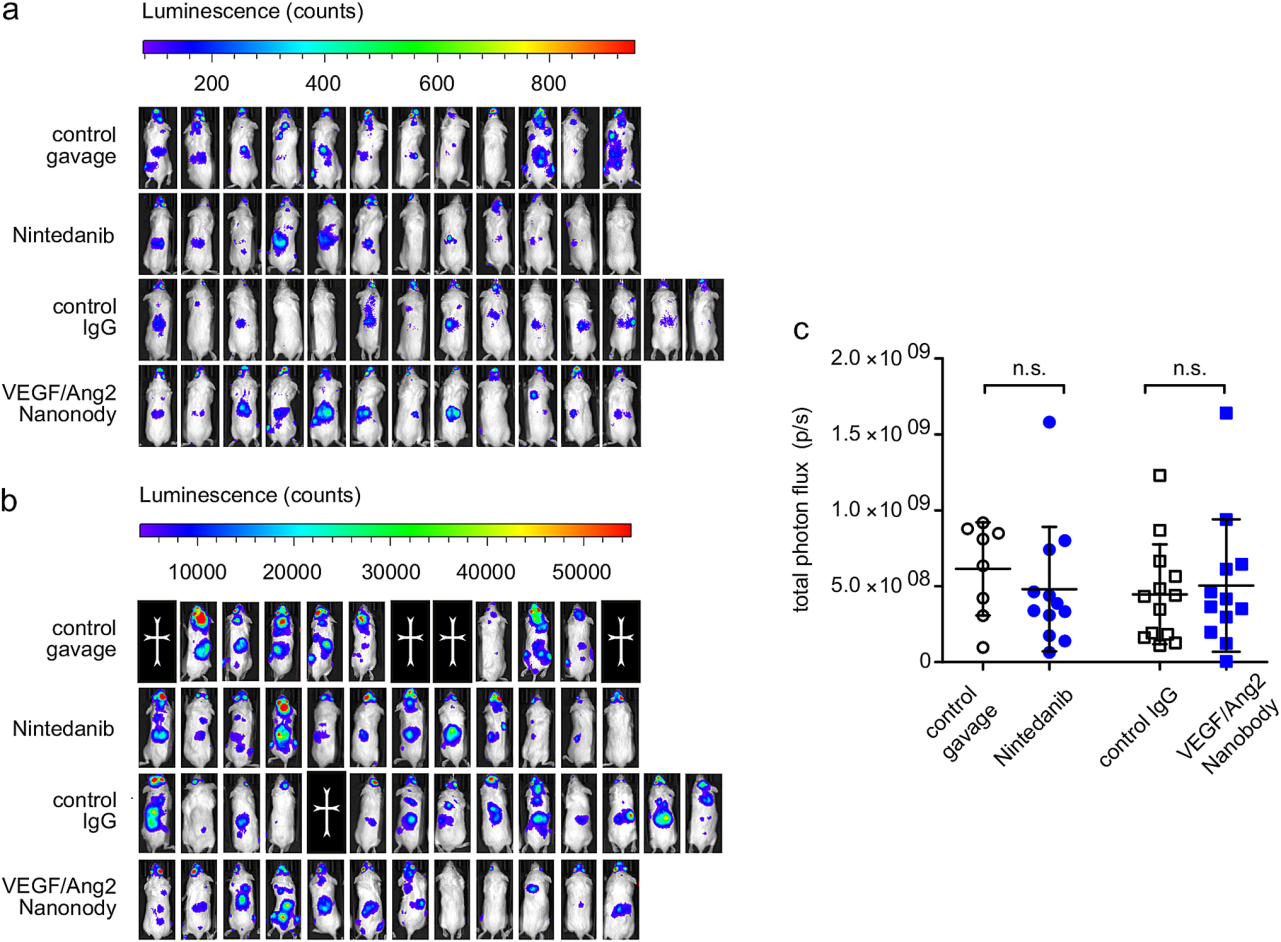
Extracranial systemic metastases are not reduced by antiangiogenic treatments. **a**, **b** IVIS luminescence images on day 14 (**a**) and day 28 (**b**) after tumor cell heart injection. Photon count visualized by heat map. **c** Scatter plot quantifying the extracranial photon flux on day 28 shows no difference between the treatment groups

Next, we sought to investigate whether the antimetastatic activity of Nintedanib and nanobody also applies to extracranial sites by using whole-body imaging (IVIS). Neither Nintedanib nor nanobody were able to reduce the burden of extracranial metastatic disease (Fig. 3, p = 0.2318 for Nintedanib, p = 0.8490 for nanobody; Mann–Whitney-U test). Furthermore, the number of extracranial metastases did not differ between treatment groups (p = 0.3797 for Nintedanib and p = 0.2084 for nanobody; Mann–Whitney-U test, data not shown). Together these data suggest that both Nintedanib and nanobody prevent metastases outgrowth in the brain, but not relevantly outside the CNS.

### Nintedanib and nanobody, but also IgG normalize blood vessels in brain metastases

**Fig. 4:**
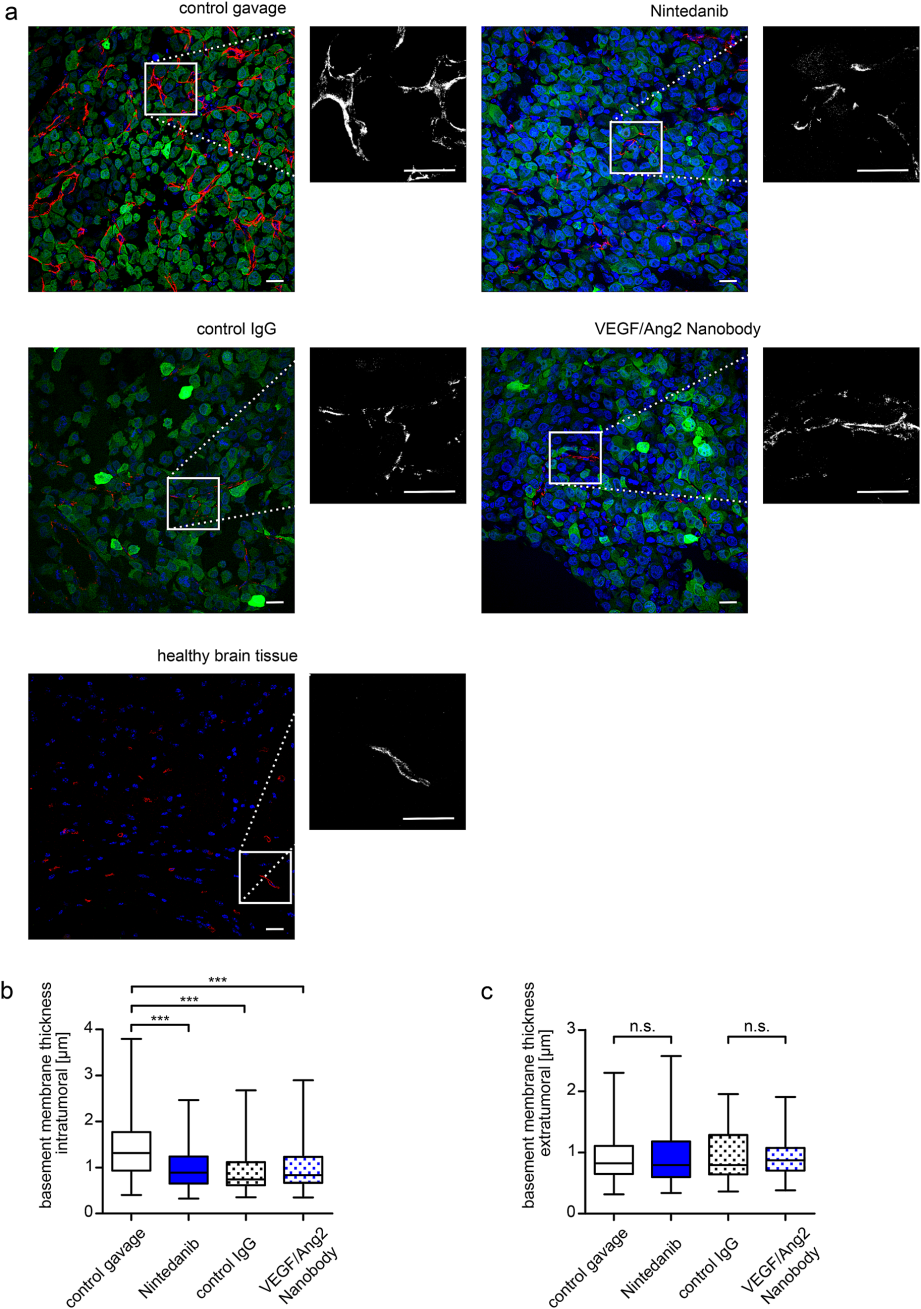
Effects of drug treatment on tumor and brain microvessels. **a** Collagen IV basement membrane staining. Confocal images of representative intratumoral regions of mice from the four treatment groups at time of death. One extratumoral image of healthy brain tissue is also displayed. Nuclear DAPI staining is shown in blue, collagen IV staining in red (Alexa Flour 546) and GFP-expressing PC14-PE6 tumor cells in green. Vascular basement membrane signal (collagen IV) is additionally shown in single-channel images. Scale bar = 20 µm, magnification 40x. Brightness adjustments were applied equally to all images. **b** Scatter plot of collagen IV immunostaining inside of metastatic brain lesions. **c** Scatter plot of collagen IV staining of healthy brain tissue. 3 regions per mouse in 3 mice per group were analyzed. ***p < 0.001, Mann–Whitney-U test. Whiskers indicate minimum and maximum values. (Color figure online)

Antiangiogenic therapies, particularly VEGF pathway inhibition, are known to reduce the abnormally thickened vascular membrane to more normal levels, which is strongly associated with improved vascular morphology and function, and finally response to irradiation in angiogenic brain tumor models [29]. To determine the vascular basement membrane thickness, we used anti-collagen IV staining as described before [29]. As expected, within metastatic foci of control mice, the vascular BM showed abnormal thickness and organization, while a very thin, linear basement membrane was found in the normal brain parenchyma (Fig. 4a). In contrast, the vascular basement membrane after Nintedanib and anti-VEGF/Ang2 nanobody treatment appeared almost normal inside of metastatic foci. (Fig. 4b, p < 0.0001 for both, Mann–Whitney-U test). Interestingly, also unspecific control IgG treatment lead to vessel normalization (p < 0.0001, Mann–Whitney-U test). In normal brain tissue, no difference between the four treatment groups was detectable (Fig. 4c).

## Discussion

In this study we examined if two antiangiogenic compounds, Nintedanib and the novel dual anti-VEGF/Ang2 nanobody BI836880, were able to prevent brain and extracranial metastases and improve survival outcomes. We found that Nintedanib and the anti-VEGF/Ang2 nanobody prolonged animal survival and reduced BM formation, while extracranial metastases were not reduced. A normalization of vascular basement membrane inside of metastatic lesions was seen in both treatment groups, but also with control IgG, which also prevented BM formation.

In accordance with the effects described for bevacizumab [15], Nintedanib and the anti-VEGF/Ang2 nanobody showed a brain-specific metastasis-preventive effect. This provides further support for the notion that antiangiogenic compounds in general might be predominantly effective in the brain, as BM, especially in lung adenocarcinoma, show a particularly stronger angiogenic reaction at this site [23, 31–33]. A recent clinical study confirms that bevacizumab, even as monotherapy (which is not relevantly active outside the brain), has a meaningful clinical activity in advanced brain metastasis, including in lung adenocarcinoma patients [34].

This specific prevention of BM was the most likely cause for the prolonged survival in the treatment groups: survival is governed by the development of BM in the used model [15]. Both Nintedanib and the anti-VEGF/Ang2 nanobody seem to have a comparable overall effectiveness when it comes to BM prevention, however showing a slightly different prevention pattern. While Nintedanib was more efficient in reducing BM volume, the dual anti-VEGF/Ang2 nanobody had a stronger preventive effect on metastasis number, which also resulted in a better survival outcome. Considering the relevance of both the early VEGF-dependent angiogenic switch and later angiogenesis-dependent growth for BM formation in lung adenocarcinoma [3, 15, 23], these findings can best be explained by an earlier activity of the anti-VEGF/Ang2 nanobody compared to Nintedanib when it comes to interference with the brain metastatic cascade.

The use of a brain seeking lung adenocarcinoma subline might have emphasized the significant survival benefit observed here. In fact, this even makes the model more realistic and relevant for the clinical situation, as under current chemotherapeutics and targeted therapies, patients with advanced lung adenocarcinoma still show an elevated incidence of BM: 42% (Taxan-based therapy) [4] to more than 50% (Crizotinib) [5] of patients develop BM within 24 months. Furthermore, the site of distant relapse in this patient population is most frequently the brain, and one third of this patient population dies because of a brain specific progress [35]. Together these facts make the development of new BM preventive treatments even more important for lung adenocarcinoma.

Unexpectedly, control IgG treatment showed similar basement membrane normalization effects as Nintedanib did, compared to control gavage treatment. Recently, syngeneic low dose IgG was shown to counteract cancer progression in melanoma, colon cancer and breast cancer mouse models, inhibiting tumor vessel proliferation and prolonging survival [36]. Anti-tumor effects of unspecific IgG have also been reported using human IgG in animal models [36, 37]. In several case reports, cancer patients who received intravenous immunoglobulin (IVIg) therapy e.g. because of a simultaneous autoimmune disease showed a significant tumor regression, too [38–40]. In our study, unspecific IgG was able to decrease the incidence of meningeal metastases, and a trend to a lower incidence of BM was observed. However, this did not translate into a survival benefit. Taken together this suggests a non-epitope specific antiangiogenic and thus metastasis-preventive mode of action of IgG, at least with respect to the formation of intracranial metastases. This could indeed be relevant for antibody therapies in general, as the ratio of given and endogenous IgG in the current study (murine IVIg/endoIgG ratio = 3.43–1.37) is comparable to the ratios of therapeutically applied and endogenous IgG during e.g. intravenous immunoglobulin therapy for several diseases in humans (IVIg/endoIgG ratio = 2.67–0.9) [41]. An anti-VEGF specific activity of IVIg has also been proposed by Damianovich et al. [42]. In a mouse model, an inhibition of melanoma and sarcoma lung metastases was shown by IVIg treatment as well as a prolongation of survival time [43]. Furthermore, it was suggested that IVIg-treatment impairs metastasis and tumor growth by promoting the M1 polarization state of tumor associated myeloid cells, which was associated with a decrease of lung metastatic foci in a melanoma mouse model [44]. Finally, it is an interesting question whether a general BM-preventive effect of the IgG molecule was the reason for the survival benefit of the intravenous anti-VEGF/Ang2 nanobody vs. the orally administered small molecule Nintedanib.

To further clarify the clinical potential of BM prevention by antiangiogenics, randomized controlled clinical trials (minimum stage II) are needed, investigating BM incidence as primary and survival and quality of life as secondary endpoints [3]. Stage III lung adenocarcinoma patients without detectable BM and no active extracranial tumor disease might be the most interesting patient population for such a study [3].

Some limitations exist. It needs to be acknowledged that the rat IgG2a used here is not the absolute appropriate control for a nanobody. Its molecular weight is about 3 times higher, since the nanobody in contrast consists of two variable antibody domains and an albumin module for half-life extension only and lacks the Fc antibody fragment [45]. However, so far there is no nanobody that would not interact with another target protein, so this was the only control compound that came into question. Moreover, a well-characterized lung adenocarcinoma cell line which is known to grow particularly angiogenesis-dependent in the brain was used in this study. The effect of an anti-VEGF/Ang2 nanobody in brain metastases on other lung adenocarcinoma cell lines needs to be evaluated. However, the simultaneous use of bevacizumab with an Ang2 inhibitor has been shown to reduce brain metastasis in the breast cancer cell lines MDA-MB-231br and 4T1br [46], so it is likely that the results of the current study can be more widely generalized to other cell lines and clinical situations. Finally, the immunodeficient mouse models that are the ones that are available today for research on the formation of blood-borne brain macrometastases [26] do not allow for conclusions about the immune microenvironment of metastatic lesions, which is another limitation of this study.

In summary, this study demonstrates that the TKI Nintedanib, an anti-VEGFA/Ang2 nanobody, but to a lesser extend also unspecific IgG can reduce BM formation, making blood vessel stabilization an attractive mechanism of BM prevention in lung adenocarcinoma. While the prime time of antiangiogenesis in oncology is fading, BM and their prevention emerge as promising targets that have not been adequately explored yet. The high effectivity against BM formation reported here also fits well to the general concept that the activity of a given drug, or class of drugs, can be very different when comparing effects in the setting of metastases prevention vs. effects on the primary tumor [13, 14, 47–50].

## Abbreviations

Ang2: Angiopoietin 2
ALK: Anaplastic lymphoma kinase
BBB: Blood brain barrier
BM: Brain metastases
BRAF: Rapidly accelerated fibrosarcoma isoform B
cMRI: Cranial magnetic resonance imaging
DAPI: 4′,6-diamidin-2-phenylindol
EGFR: Epidermal growth factor receptor
GFP: Green fluorescent protein
IVIg: Intravenous immunoglobulin
IVIS: In vivo bioluminescence imaging system by Perkin Elmer
MEK: Mitogen-activated protein kinase kinase
ROI: Region of interest
RTG: Research training group
TKI: Tyrosine kinase inhibitor
VEGF: Vascular endothelial growth factor
